# The Diagnostic Value of the Thermostatic Amplification of Ribonucleic Acid in Bronchoalveolar Lavage Fluid in Smear-Negative Pulmonary Tuberculosis

**DOI:** 10.3389/fpubh.2022.830477

**Published:** 2022-06-16

**Authors:** Zhengxing Wu, Jichan Shi, Yueying Zhou, Ning Pan, Chaochao Qiu, Lianpeng Wu, Xiangao Jiang

**Affiliations:** Department of Infection Disease, Wenzhou Central Hospital, The Dingli Clinical Institute of Wenzhou Medical University, Wenzhou, China

**Keywords:** smear-negative pulmonary tuberculosis, bronchoalveolar lavage fluid, RNA simultaneous amplification, testing, diagnostic value

## Abstract

**Objective:**

This study aimed to determine the value of the simultaneous amplification and testing for *Mycobacterium tuberculosis* in bronchoalveolar lavage fluid (BALF) in the diagnosis of smear-negative pulmonary tuberculosis (PTB).

**Methods:**

A total of 316 patients were selected, of which 197 had smear-negative PTB (observation group), and 119 did not have TB (control group). Bronchoscopy was performed in both groups, and BALF samples were collected for acid-fast bacilli smears, simultaneous amplification/testing for TB (SAT-TB), and BACTEC MGIT 960 cultures. The sensitivity, specificity, positive predictive, and negative predictive values of SAT-TB in BALF for the diagnosis of negative TB were calculated.

**Results:**

The sensitivity of SAT-TB detection was 45.18%, which was significantly higher than smears and slightly lower than cultures. The specificity of SAT-TB was 99.16%, which differed slightly from the other two methods. The positive predictive value was 98.89%, which was not significantly different from the other two methods. The negative predictive value of SAT-TB was 58.91%, which was higher than smears and slightly lower than cultures.

**Conclusion:**

The very high specificity and negative prediction of SAT-TB in BALF means that the method has great application value for the rapid diagnosis of smear-negative PTB.

## Introduction

Tuberculosis (TB) is a lung disease caused by *Mycobacterium tuberculosis* (Mtb). Currently, it is one of the most harmful chronic infectious diseases worldwide, especially in developing countries. According to the WHO's statistics, two billion people are currently infected with TB globally, with 8.9 million new cases and 1.6 million deaths each year (1). The number of new patients with active TB in China in 2019 ranked third in the world (2).

At present, the diagnosis methods for pulmonary TB (PTB) mainly include clinical manifestations, imaging, sputum smear microscopy, culture methods, immunology, and molecular biology diagnostics (3). Since the symptoms, signs, and X-ray indexes of TB are similar to many lung diseases, a differential diagnosis is required. Currently, a definite diagnosis of TB is obtained by detecting smear-positive and sputum-positive TB. However, the rate of conventional smear-positive detection is low (10.0%−30.0%), and the sputum culture takes 45 days, which poses difficulties for the rapid diagnosis of the disease (4).

Many molecular biology techniques are available for the rapid diagnosis and identification of Mtb (5). The traditional testing methods of amplifying DNA produce false positives and false negatives, which is challenging for clinical diagnosis (6). In recent years, simultaneous amplification testing for Mtb (SAT-TB) has begun to be used diagnostically since it can quickly and accurately determine the presence of Mtb in untested samples (7).

In this study, we used the SAT technique to detect Mtb ribonucleic acid (RNA) in the bronchoalveolar lavage fluid (BALF) specimens of patients with suspected TB; these were compared with a rapid TB culture to explore the diagnostic value of SAT-TB for smear-negative PTB.

## Materials and Methods

### General Data

The ethics committee of Wenzhou Central Hospital collected and approved a total of 316 outpatients and hospitalized patients with suspected TB from 1 January 2019 to 1 February 2021. Patients who met the following criteria were recruited: (1) pulmonary shadows on chest X-rays and clinically suspected TB, and (2) smear-negative with acid-fast staining three consecutive times. The exclusion criteria included the following: (1) BALF samples were smear-positive with acid-fast staining, and (2) no positive findings with clinical bronchoscopy. According to the diagnostic criteria of PTB (8), a total of 316 cases were divided into the smear-negative group (the observation group; *n* = 197) and the non-TB group (the control group; *n* = 119). The clinical or pathological results were diagnosed as tumors, inflammation, or other diseases. See Table 1 for the general information on the age and gender of the patients in the two groups.

**Table 1:** Comparison of baseline data between the two groups.

**Group**	***n***	**Gender (** ***n*** **)**		**Age (year)**	
		**Male**	**Female**	**Range**	**Average age**
Observation	197	132	67	11–81	40.00 ± 16.75
Control	119	83	36	28–86	52.17 ± 15.70
*F/χ^2^*	–	1.285		1.547	
*P*-Value	–	0.082		0.091	

### Methods

Fibreoptic bronchoscopy was performed on all patients in combination with BALF antiacid staining, SAT-TB, and rapid TB culture. All study subjects were informed and agreed to the above examinations.

(1) Collection of BALF: Bronchoscopy was performed on all study subjects, and specimens containing 5–10 ml of BALF were collected. The BALF specimens above 5 ml were taken and mixed with 0.5% *N*-acetyl-l-cysteine-NaOH digestion solution according to the turbidity for vortex concussion (the liquefaction time did not exceed 5 min); then, they were centrifuged at 3,000 rpm for 20 min. The precipitation was washed with 6.8% phosphate-buffered saline.
(2) Ziehl–Neelsen staining: The 5-ml BALF specimens were placed in centrifuge tubes and centrifuged at 3,000 rpm for 20 min. The supernatant was removed, and the sediment smear was taken and dried naturally. Then, it was heated and fixed by an alcohol lamp for anti-acid staining.
(3) BACTEC MIGT 960 liquid culture for TB: The BACTEC MIGT 960 mycobacterial analysis system (produced by the American BD Corporation) and its accompanying MIGT culture tubes were used according to the kit instructions. A 0.5-ml volume of precipitate was withdrawn with a disposable sterile straw after washing with the BALF treatment liquid. It was added to the MGIT culture tube, covered with the MIGT tube lid, mixed well, and put into the apparatus for incubation.
(4) Detecting BALF with SAT-TB: After washing, digestion, and centrifugation with the BALF treatment solution, 50-μl RNA lysis buffer was added. It was sonicated for 15 min (power 300 W) and centrifuged at 13,000 rpm for 5 min. Then, the supernatant was used as the amplification template. An Mtb nucleic acid detection kit (RNA constant-temperature amplification) (trade name: SAT-TB, Shanghai Renhua Biotechnology Co., Ltd.) was used according to the kit instructions.

### Statistical Analysis

Statistical analysis was performed using SPSS 25.0 software. The Wilcoxon rank-sum test was used to compare age differences between groups, and the chi-squared test was adopted to compare gender differences between groups. The sensitivity and specificity of the three tests were calculated using independent sample *t*-tests. Pairwise comparisons of the sensitivity of the three detection methods were performed using the chi-squared test. Comparisons of the lavage RNA/TB smears and RNA/TB cultures were performed using Fisher's exact probability test. The comparison of the lavage TB smears and cultures was not possible using Fisher's exact probability test because both groups were 100%. The confidence intervals of the rate differences were calculated using the corrected Newcombe–Wilson method along with the positive and negative prediction values. A value of *P* < 0.05 was statistically significant.

## Results

### Basic Information of Both Groups

All 197 patients in the observation group had secondary TB, including 12 cases of drug-resistant TB and one case of non-Mtb pulmonary disease. Of the 119 patients in the control group, 66 had pneumonia, 23 had non-Mtb pulmonary disease, 24 had obsolete TB, two had silicosis, two had pulmonary mycosis, and two had lung cancer.

### Diagnostic Value of the Simultaneous Amplification and Testing of Bronchoalveolar Lavage Fluid for Smear-Negative Pulmonary Tuberculosis

Clinical diagnosis was used as the positive criterion for diagnosing PTB. The clinical diagnosis of TB includes culture-positive and culture-negative TB, and the number of non-TB cases is the negative standard. Of the lavage patients who were TB positive in the non-TB group, 82 were SAT-TB positive, 31 were SAT-TB negative, and 1 was SAT-TB positive. The sensitivity of the BALF SAT-TB test was 45.18%, with 99.16% specificity, 98.89% positive predictive value, and 52.21% negative predictive value, as shown in Table 2.

**Table 2:** SAT-TB test of BALF for diagnosis of smear-negative pulmonary tuberculosis.

	**Pulmonary tuberculosis group**		**Non- tuberculosis group**
	**Culture positive (definite diagnosis)**	**Culture negative (clinical diagnosis)**	
SAT-TB positive	82	8	1
SAT-TB negative	31	76	118
Total	113	84	119

### Comparison of the Three Detection Methods for Bronchoalveolar Lavage Fluid Specimens

Clinical diagnosis is used as the positive standard to diagnose PTB. The clinical diagnosis of TB includes culture-positive and culture-negative TB, with the number of non-TB cases used as the negative standard. The diagnostic value of the smear, culture, and SAT-TB methods in smear-negative PTB was investigated. As shown in Table 3, the sensitivity of SAT-TB detection was 45.18%, which was significantly higher than the smear method and slightly lower than the culture method. The specificity of SAT-TB was 99.16%, which was slightly different from the other two methods. The positive predictive value was 98.89%, which was not significantly different from the other two methods. The negative predictive value of SAT-TB was 58.91%, which was higher than the smear method and slightly lower than the culture method.

**Table 3:** Accuracy of the three test methods for diagnosis of smear-negative tuberculosis.

**Test methods**	**Sensitivity**	**Specificity**	**Positive predictive value**	**Negative predictive value**
Smear	8.12%	100%	100%	39.67%
Culture	57.65%	100%	100%	58.91%
SAT-TB	45.18%	99.16%	98.89%	52.21%

## Discussion

Simultaneous amplification/testing for TB is a new generation of nucleic acid detection technology based on RNA constant-temperature amplification and real-time fluorescence detection technologies. The detection target was Mtb-specific 16S rRNA. At the same temperature (42°C) and using RNA as the starting template, a double-stranded DNA copy was produced by MMLV reverse transcriptase. Then, T7 RNA polymerase was used to produce multiple RNA copies from the DNA copy.

Each RNA copy starts from reverse transcription and enters the next round of the amplification cycle. At the same time, the fluorescent labeled probe specifically binds to these RNA copies to produce fluorescence. The fluorescence detector can capture the fluorescence signal in real-time, which directly reflects the generation of amplification products to quickly and accurately determine whether Mtb is present in the sample to be tested (9).

Clinicians have gradually recognized RNA detection because of its high sensitivity, its ability to distinguish between dead and living bacteria, and its easy degradation of RNA, which makes it difficult to contaminate (10, 11). The advantage of SAT-TB detection technology is the constant-temperature amplification at 42°C, and its initial target and amplification product is RNA. The quantitative detection of RNA can directly reflect the presence of live bacteria in the tested sample (10).

The pathological testing of sputum in the current diagnostic criteria for TB plays a decisive role. Smears for acid-fast bacilli are affected by the specimen material, the patient's intermittent discharge of bacteria, the number of bacteria in the specimen, and many other factors, resulting in low sensitivity. A smear-positive result requires more acid-fast bacilli, and its sensitivity is limited to over 10,000 biological/ml in sputum. However, it may also be caused by non-Mtb (12). In this study, although the specificity for acid-fast bacilli smears was 100%, the sample size was not large enough to cover patients with non-Mtb who were acid-fast bacilli smear-positive.

The results of the fifth national TB epidemiological sample survey in 2010 (13) revealed that the prevalence rate of smear-negative TB accounted for 85.62% of patients with TB, which is the top priority in TB prevention and control work. Currently, Mtb culture remains the gold standard for diagnosing active TB. However, it usually takes 3–8 weeks for a diagnosis. The improved BACTEC rapid culture system allows the culture time to be as fast as 15 days, although there is still a certain gap in clinical requirements for the rapid diagnosis of TB. Due to delayed diagnoses, these patients have waited longer than patients with sputum smear-positive results, leading to an increase in misdiagnoses and transmission rates (14).

This study shows that SAT-TB has high positive predictive values (98.89%) and can be completed within 120 min. When BALF samples are positive, regardless of smear results, it is recommended that patients with clinically suspected TB are administered anti-TB treatment promptly without waiting for the culture results (6–8 weeks) (15). Positive results from SAT-TB could exclude a single infection with non-Mtb (NTM). One patient in this study had a mixed infection involving Mtb and NTM, which indicates that SAT-TB cannot detect a mixed infection in the same sample. This study also showed that the specificity of the SAT-TB was 99.16%. Based on the higher SAT specificity, the patient was most likely to be infected with Mtb when both the SAT and smear were positive.

At the same time, the real-time amplification signal Ct values generated by SAT-TB can be used for the relative quantification of TB bacteria, which may contribute to the clinical management of TB treatment. The continuous dilution of Mtb indicated that SAT-TB detection had high sensitivity with a detectable 100 CFU/ml (16). Of the 119 patients without TB who were included, 22 with obsolete TB tested negative for SAT-TB, indicating that this test could evaluate the evolving trend of patients with TB.

This study showed that the SAT-TB diagnostic sensitivity of BALF in patients with smear-negative results was 45.18%, and the negative predictive value was 52.21%, which was lower than the findings of Fan et al. (17). The reasons affecting the efficiency of SAT-TB detection were as follows: (1) Unequal distribution of the test suspension and the presence of enzyme amplification inhibitors (18, 19). (2) Specimens were placed for a long time. This test is a delivery project. The number of patients examined daily in our hospital is very high, and the time between removing BALF from the microscope and maintaining it at room temperature may be more than 5 h. Some specimens still need to be tested in the laboratory refrigerator. The long placement time may lead to a reduced detection rate of 16S rRNA in SAT-TB specimens. (3) The study's focus limited clinical bronchoscopy manipulation. Our study was designed to include patients with suspected active TB with negative tests for acid-fast bacilli, but the lesions in these patients were generally mild and peripherally limited, and the patients often had little or even no sputum. Some bronchial lavage operations did not fully reach the infected lesion, resulting in a small number of mycobacteria. Furthermore, the small volumes of BALF tested will also impact the positive rate.

In the environment, RNA is unstable, and it is easily degraded. Although contamination was effectively avoided, one of the 119 patients without TB in this study remained false positive for the SAT-TB of BALF, suggesting that in the process of endoscopic disinfection and BALF retention, specimen packaging/delivery, and laboratory operation, specimen contamination still occurred. In the clinical operation of this technology, the operating specifications should be strictly followed to prevent the impact of human factors from reducing its testing efficiency.

In conclusion, although this study showed that the sensitivity of SAT-TB is lower than in studies at home and abroad for various reasons, and its low negative predictive value limits its usefulness in excluding PTB diagnosis, SAT-TB-negative specimens still require routine culture tests and specifications. Furthermore, current techniques cannot prevent the occurrence of false-positive and false-negative results. Further multicenter and large cohort studies are needed to corroborate the results of this study.

The use of SAT-TB technology can aid the early detection and diagnosis of TB. The technology has high specificity, low cost, and rapid detection, and it is undoubtedly a major benefit for patients with smear-negative TB. From the perspective of public health, it also significantly reduces the overall liquidity and mortality of TB in China and has great application value.

## Data Availability Statement

The original contributions presented in the study are included in the article/supplementary material, further inquiries can be directed to the corresponding author.

## Ethics Statement

The studies involving human participants were reviewed and approved by Wenzhou Center Hospital. The patients/participants provided their written informed consent to participate in this study.

## Author Contributions

ZW and JS conceived of the study. YZ, NP, CQ, and LW participated in its design and coordination. XJ helped to draft the manuscript. All authors read and approved the final manuscript.

## Funding

This work was funded by Wenzhou Science and Technology Plan Project (Y2020277) and Medical and Health Project of Wenzhou Major Scientific and Technology Innovation (2020ZY2020019).

## Conflict of Interest

The authors declare that the research was conducted in the absence of any commercial or financial relationships that could be construed as a potential conflict of interest.

## Publisher's Note

All claims expressed in this article are solely those of the authors and do not necessarily represent those of their affiliated organizations, or those of the publisher, the editors and the reviewers. Any product that may be evaluated in this article, or claim that may be made by its manufacturer, is not guaranteed or endorsed by the publisher.
